# Occupational physical behaviors and knee pain among eldercare workers: A prospective accelerometer study

**DOI:** 10.5271/sjweh.4260

**Published:** 2026-05-01

**Authors:** Sebastian Venge Skovlund, Christian Tolstrup Wester, Stavros Kyriakidis, Luiz Augusto Brusaca, Lars Louis Andersen, Emil Sundstrup, Charlotte Diana Nørregaard Rasmussen

**Affiliations:** 1 National Research Centre for the Working Environment, Copenhagen, Denmark.; 2 Department of Sports Science and Clinical Biomechanics, University of Southern Denmark, Odense, Denmark.; 3 Laboratory of Clinical and Occupational Kinesiology, Department of Physical Therapy, Federal University of São Carlos, São Carlos, São Paulo, Brazil.; 4 Department of Occupational Health, Psychology and Sports Sciences, University of Gävle, SE-801 76 Gävle, Sweden.

**Keywords:** accelerometry, compositional data analysis, musculoskeletal disease, worker, workplace

## Abstract

**Objective:**

The aim of this study was to explore the prospective association between compositions of accelerometry-measured occupational physical behaviors and the risk of knee pain among eldercare workers.

**Methods:**

We performed a prospective study among 377 eldercare workers employed across 20 Danish nursing homes. Occupational physical behaviors were measured using thigh-worn accelerometers over 1–4 working days. Workers reported intensity of and days with knee pain in a questionnaire at baseline and after one year. We explored associations between compositions of occupational physical behaviors [ie, sedentary, standing, light physical activity (LPA), and moderate-to-vigorous physical activity (MVPA)] and knee pain, adjusting for potential confounders.

**Results:**

No significant associations were found. Trends were found for increased occupational time spent in MVPA and decreased risk of days with knee pain [relative risk (RR) 0.58, 95% confidence interval (CI) 0.32–1.05, P=0.07] in main analyses, and for decreased risk of knee pain intensity among non-knee pain cases (RR 0.36, 95% CI 0.12–1.13, P=0.08) in sensitivity analyses.

**Conclusions:**

No significant associations were found between baseline occupational physical behaviors and knee pain at one-year follow-up. However, a non-significant trend suggested that increasing occupational MVPA might be associated with reduced risk of knee pain at follow-up, though studies with larger samples are needed to confirm this finding.

Musculoskeletal disorders of the knee (knee disorders) are a prevalent health condition with significant implications for individuals, workplaces, and societies as a whole (1–3). The knee pain often associated with knee disorders can be caused by specific musculoskeletal diagnoses (eg, knee osteoarthritis) or be non-specific (ie, of unknown cause). Both studies on non-specific knee pain and knee osteoarthritis will be cited throughout this paper. Knee osteoarthritis remains the most prevalent musculoskeletal condition causing (specific) pain in the knees (1) and is expected to increase in the years to come due to, eg, changing demographics and population aging (4, 5). Importantly, knee disorders are also highly prevalent in the working population (3, 5). Specifically, Valter and colleagues reported a prevalence of severe non-specific knee pain of 11% among 75 000 currently employed workers, with about 15% of workers >50 years affected (3).

Healthcare workers employed in eldercare are an example of an occupational group at high risk of non-specific knee pain (6). Furthermore, non-specific knee pain has been found to be a significant risk factor for leaving nursing care, sickness absence, and disability pension among healthcare workers (6–8). In light of the current and future prevalence of knee disorders such as knee osteoarthritis, and the challenges pertaining to labor shortage that the healthcare industry and the society as a whole are facing (9), identifying occupational risk factors and protective factors as well as development and implementation of effective preventive interventions are highly relevant.

High occupational physical workload has been suggested to be a risk factor for non-specific knee pain (10–13) and knee osteoarthritis (14–16). For instance, high exposure to occupational behaviors such as standing, walking, and lifting, as well as occupational postures like kneeling or squatting have been associated with an increased risk of knee osteoarthritis (14–17). Eldercare work typically comprises long durations of standing (eg, while assisting the residents), walking, and lifting (eg, resident handling) (18), which may thus increase the risk of knee disorders. Nonetheless, the majority of the evidence linking occupational physical workload to knee disorders relies on self-reported information, which are more prone to bias compared to technical measures of occupational physical workload using, eg, accelerometers (19–21).

Recently, Locks and colleagues (17) reported temporal patterns of accelerometry-measured occupational standing, ie, frequent short bouts of occupational standing, to be associated with increased non-specific knee pain intensity in a cross-sectional study. To our knowledge, the *prospective* risk of knee disorders in general, or specifically knee pain associated with technically measured occupational physical workload is yet to be investigated. In addition, many studies, including the one by Locks et al (17), do not take the compositional nature of time-use data into account. In reality, the total time per day is finite, whereby increasing time in one physical behavior, eg, occupational walking, will inevitably lead to less time spent in one or several other mutually exclusive physical behaviors, eg, occupational sitting. Therefore, a methodological approach with a 24-hour perspective taking the co-dependency of physical behaviors into account is suitable. Compositional data analysis (CoDa) is one such approach (22, 23), which has previously been applied in occupational health studies investigating musculoskeletal pain as an outcome (18, 24, 25), however, no studies have examined knee disorders specifically.

This prospective study aimed to investigate the association between the composition of accelerometry-measured occupational physical behaviors and the risk of non-specific knee pain over one year among eldercare workers.

## Methods

### Study design and setting

This study used data from the Danish observational study of eldercare work and musculoskeletal disorders (DOSES), a prospective study among eldercare workers described elaborately elsewhere (26). Data were collected from September 2013 to January 2016. DOSES is a workplace study designed to examine associations between physical and psychosocial working conditions, musculoskeletal pain and its consequences among eldercare workers at Danish nursing homes. This study is reported following the STROBE reporting guidelines for cohort studies (27). The Danish Data Protection Agency and the Ethics Committee for the Capital Region of Denmark granted ethical approval (H-4-2013-028), and written informed consent was obtained from all eldercare workers prior to their participation in the study.

### Study population

Eighty-three nursing homes located in Zealand in the greater Copenhagen area were invited to participate in the study with the aim of including various nursing home sizes and care models. Eligibility criteria for participation included: (i) aged 18–65 years; (ii) employed >15 hours per week; (iii) working day, evening, or rotational shifts; and (iv) allocation of ≥25% of working time on activities related to direct resident care.

**Figure 1:**
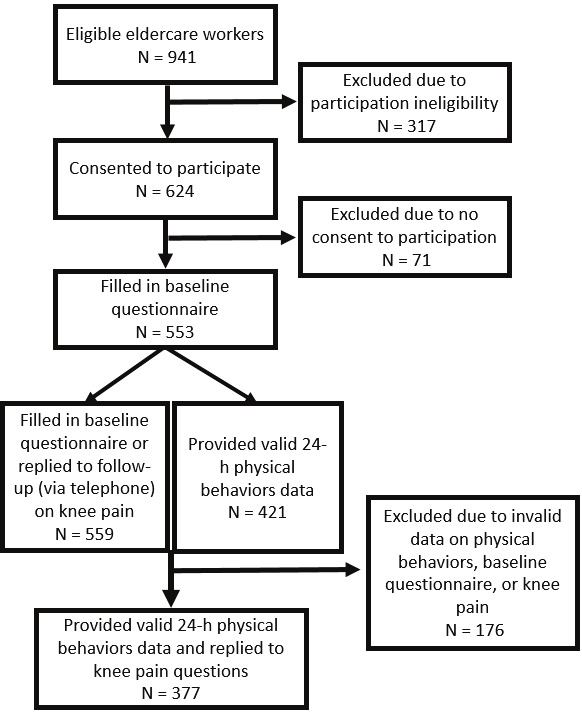
Flowchart of participants.

Workers were excluded from the study if they were on long-term sick leave, pregnant, had a temporary employment, or spent <25% of their total working time on tasks related to the direct care of the elderly residents (eg, more administrative workers spending the majority of their working time on non-care related tasks) (26). Twenty nursing homes (18 municipal and 2 private), including 126 wards and 941 eligible eldercare workers, agreed to participate (figure 1).

### Knee pain (outcome)

Self-reported non-specific knee pain was assessed by the number of days with – and intensity of – pain in the knee and was reported at baseline and after one-year follow-up (via telephone) using the Nordic Musculoskeletal Questionnaire (28). Workers were asked about the number of days with knee pain in the previous month: “In the last four weeks, how many days did you have pain in your knees?”, with possible answers of 0–28 days. If the workers replied having >0 days of pain, they were asked to rate the intensity of knee pain on a 11-point numerical rating scale (NRS): “On a scale of 0–10, what was the worst pain you experienced in your knees within the past four weeks?”, with answers ranging from 0=no pain to 10=worst possible pain.

### Accelerometer measurements of physical behaviors (exposure)

We assessed physical behaviors at baseline using data from one tri-axial ActiGraph GT3X+ accelerometer (Actigraph, Pensacola, FL, USA) placed on the right thigh. All participants were asked to wear the accelerometer without dismantling it for ≥4 consecutive days, including ≥2 workdays. Participants were also instructed to keep a diary of their sleep and wake times (time in/out of bed), the start and end times of their work shifts, whether they had a day off work, and any instances when the accelerometer was removed, including the time and reason for its removal. The accelerometer data were downloaded using the Actilife Software version 5.5 (Actigraph, Pensacola, FL, USA) and processed using a custom-made MATLAB program, Acti4 (29, 30), which has been shown to classify different behaviors (ie, postures and activities) with a high sensitivity and specificity.

Based on Acti4 output, all non-workdays as well as non-wear periods were excluded. Then, we identified four mutually exclusive behaviors (“compositional parts”) that completely accounted for time used during work and leisure-time hours, ie, sedentary (sitting and lying), standing (standing still and moving [ie, occasional movements in an upright position]), light physical activities (LPA; slow walking), and moderate-to-vigorous physical activities (MVPA; fast walking, stair climbing, running, and cycling) (18). Time-in-bed (a proxy for sleep), identified based on the diary information, was added to the dataset to arrive at a full 24-hour physical behavior composition. Time-in-bed data were checked for accuracy by visual inspection of the accelerometer data using Acti4. Only workers who had ≥1 day with valid work and leisure-time data were included for further analysis. Thus, a valid period of work and leisure time was defined as wear time of ≥4 hours or comprising ≥75% of the average wear-time across days (31). Finally, daily time spent in each behavior during work and leisure time was averaged over all available workdays for each worker.

### Confounders

Potential confounders were chosen *a priori* based on data availability, previous literature, and theoretical assumptions of the possible associations with knee disorders and occupational physical behaviors. Information on the following chosen confounders were collected at baseline via a questionnaire or a health check (including pre-baseline screening questionnaire): age (years), gender (male or female), body mass index (BMI, kg/m^2^; calculated from measured height and body weight), smoking status, self-rated general health, knee pain intensity (collected pre-baseline, see below), influence and social support at work, and physical activity during leisure. Smoking status was dichotomized into smokers (“yes, daily”, “yes, once in a while”) and non-smokers (“no, but smoked in the past”, “no, I have never smoked”). Self-rated general health was obtained by the question “In general, how would you rate your health?” with possible replies “excellent”, “very good”, “good”, “fair” or “poor” (32). Self-reported information on worst knee pain intensity during the past four weeks (0–10 NRS) was obtained in the screening questionnaire collected chronologically prior to the baseline questionnaire [see protocol for more details (26)]. Physical behaviors during leisure time were measured using accelerometers, as described above. Workers rated their degree of influence at work (two items: IN1 and IN3) and social support at work (three items: SC1, SC2, and SC3), using 5-point Likert scales derived from the Copenhagen Psychosocial Questionnaire (33). Answers to these five psychosocial questions were converted into a 0–100 scale before averaging items within each dimension, with higher scores reflecting better psychosocial working conditions (33).

### Statistical analysis

Occupational and leisure-time physical behavior data were presented in minutes and percentage of time spent per day using arithmetic means and standard deviation (SD).

To investigate the association between occupational physical behaviors and knee pain, we initially processed the physical behavior data using a compositional data analysis (CoDA) approach (23). In brief, occupational and leisure-time physical behaviors are inherently interdependent and constrained by the fixed 24-hour day, meaning that increasing time spent on one behavior will result in a reduction in time allocated to other mutually exclusive behaviors. Thus, it is recommended to apply CoDA-based methods for analyzing such data (23).

Following the recommended CoDA approach (23), the four-part time composition of work (sedentary, standing, LPA, and MVPA) and the five-part time composition of leisure time (sedentary, standing, LPA, MVPA, and time-in-bed) were transformed into sets of isometric log-ratio (ilr) coordinates (22). In this data transformation, work and leisure-time physical behavior compositions were treated as two separate sub-compositions of a 24-hour day (23, 34) as the purpose of the study was to examine the association of occupational physical behaviors with knee pain. Additionally, as only the first ilr coordinate is interpretable (22, 23), and to enable the examination of each occupational physical behavior (ie, sedentary, standing, LPA, and MVPA) in relation to knee-pain outcomes, a pivoting method was implemented. In this method, the first behavior in the equation was rotated each time and the set of ilr coordinates was calculated, allowing each occupational behavior to be expressed relative to the remaining occupational behaviors (22).

After the ilr transformation, a separate negative binomial generalized linear mixed-effects model was fitted for each of the occupational ilr coordinates (crude model) to examine the longitudinal association with number of days with knee pain and the intensity thereof. In the adjusted models, we included the above-mentioned confounders. To account for the nested structure of the data (workers within wards, and wards within nursing homes), the model included random effects for workers, wards, and nursing homes. Model diagnostics were conducted using a likelihood ratio test to check for over-dispersion, as well as assessing influential observations and verifying the homoscedasticity of the deviance residuals. Effect estimates were expressed as relative risk (RR) with 95% confidence intervals (CI). Additionally, the significance of the overall occupational composition was evaluated using Wald chi-squared tests through analysis of variance (ANOVA) tables, applying type II tests. The significance level was set at P<0.05.

The regression coefficients of the ilr coordinates obtained from the negative binomial generalized linear mixed-effects models were expressed on a logarithmic scale, allowing only the interpretation of the direction of the associations. We applied compositional isotemporal substitution analysis to illustrate hypothetical changes in the RR when reallocating time between occupational behaviors (35).

Firstly, a reference composition was calculated, ie, the sample’s compositional mean of all occupational physical behaviors (table 1). Secondly, based on the calculated reference composition, new hypothetical compositions of occupational physical behaviors were generated by using two methods, ie, one-to-remaining and one-to-one reallocations. For the one-to-remaining reallocations, new theoretical compositions were generated by incrementally increasing/decreasing time spent in each occupational physical behavior by decreasing/increasing time spent in the remaining occupational physical behaviors in 5-minute increments according to their proportions while maintaining the total occupational composition constant (35). For the one-to-one reallocations, new theoretical compositions were generated by reallocating 5-minute increments from one occupational physical behavior to another, while keeping the remaining occupational behaviors constant.

**Table 1:** Baseline characteristics. [SD=standard deviation; BMI=body mass index; LPA=light physical activity; MVPA=moderate-to-vigorous physical activity; NRS=numerical rating scale].

Variable		N	%	Mean (SD)
Age (years)				45.8 (10.5)
Gender				
	Women	360	95.5	
	Men	17	4.5	
Ethnicity				
	Denmark	300	79.6	
	Other country	77	20.4	
Job seniority (months)				197.4 (132.7)
BMI (kg/m^2^)				26.4 (5.3)
Smoking				
	Non-smoker	239	63.4	
	Smoker	138	36.6	
Position				
	Care assistant	155	41.1	
	Care helper	171	45.4	
	Other	51	13.5	
Shift				
	Day	223	59.2	
	Evening	78	20.7	
	Day / evening	18	4.8	
	Day & evening / night	58	15.4	
Working hours				32.4 (3.6)
Leadership quality (0–100)				60.5 (17.2)
Collegial support (0–100)				71.7 (14.8)
Influence at work (0–100)				57.1 (18.8)
Knee pain				
	Days (baseline)			4.9 (8.7)
	Days (1-year follow-up)			4.4 (8.1)
	≥1 day (baseline)	216	57.3	
	≥1 day (follow-up)	178	62.5	
	Intensity (baseline, 0–10 NRS)			2.1 (2.9)
	Intensity (1-year follow-up, 0–10 NRS)			1.8 (2.7)
Accelerometry				
	Measurement days			2.7 (1.2)
	Measurement hours (all activities and sleep included across domains)			1391.6 (97.4)
Total worktime (minutes per day)				
	Worktime per day			436.5 (68.5)
	Sedentary		37.6	165.7 (58.2)
	Standing		46.1	198.2 (52.7)
	LPA		2.3	11.4 (7.0)
	MVPA		14	61.1 (19.9)
Total leisure time (minutes per day)				
	Leisure time (all activities except sleep)			535.0 (72.4)
	Sedentary		34.5	329.0 (83.0)
	Standing		14.9	146.3 (50.3)
	LPA		0.9	10.2 (6.3)
	MVPA		4.8	48.8 (23.3)
	Sleep		45	420.1 (63.9)

The new hypothetical compositions were then transformed into ilrs following the procedure described earlier. For these predictions, the numeric covariates used in the models were held at their mean values, while for the categorical variables, the most prevalent category was applied. The parameter estimates from the negative binomial generalized linear mixed-effects models were utilized to predict the RR for the new hypothetical compositions. These predictions indicate the estimated differences in the risk of knee pain outcomes compared to the reference composition (36). The bootstrapping (R=1000) method was used to compute the 95% CI for the model based on negative binomial generalized linear mixed-effects models. In addition, we performed sensitivity analyses separately for (a) knee pain cases (knee pain intensity ≥3) and (b) non-knee pain cases (knee pain intensity <3 out of 10) to test whether baseline knee pain intensity affected the association between occupational physical behaviors and risk of knee pain.

All statistical analyses were carried out using *R* v4.3.3 (R Foundation for Statistical Computing, Vienna, Austria). Specifically, CoDA data processing was performed using the package ‘compositions’ v2.0-2 (37).

## Results

### Baseline characteristics

Baseline characteristics of the sample are provided in table 1. Valid data on occupational physical behaviors and knee pain from 377 workers from 20 Danish nursing homes were included in the final analysis (figure 1). Participants were predominantly female (95.5%), with a mean age of 45.8 years. At baseline, 216 (57.3%) out of 377 reported ≥1 day of knee pain, and at follow-up, 178 (62.5%) out of 285 reported ≥1 day of knee pain.

Occupational physical behaviors data were available for an average of 2.7 (SD 1.2) days and a total of 1391.6 (SD 97.4) hours from the included eldercare workers, including 955.1 (SD 79.2) hours of leisure time including sleep, and 436.5 (SD 68.5) hours of worktime.

### Main analyses

*One-to-remaining reallocations.* Results from our main analyses are reported in table 2, table 3, and figure 2A–B. Table 2 indicates that the overall composition of physical behaviors at work was not statistically significantly associated with either days with knee pain (P=0.31) or knee pain intensity (P=0.53).

**Table 2:** Estimates of the overall composition as a predictor for days with knee pain and knee pain intensity. [Pr(>Chi^2^)=P-value for Chi^2^].

Knee pain outcomes	Univariate analysis			Multivariate analysis	
	Chi^2^	Pr(>Chi^2^)		Chi^2^	Pr(>Chi^2^)
Days	1.54	0.67		3.57	0.31
Intensity	1.26	0.74		2.22	0.53

**Table 3:** Regression relative risk (RR) estimates obtained from the generalized linear mixed-effects models to investigate the association between occupational physical behaviors (sedentary, standing, light physical activity, and moderate-to-vigorous physical activity) and risk of days with knee pain and knee-pain intensity. [95% CI=95% confidence interval; iIr=isometric log-ratio; LPA=light physical activity; MVPA=moderate-to-vigorous physical activity.] Example of how to interpret occupational composition ilr1 RR: A one-unit increase in the log of time spent in occupational MVPA relative to time spent in the remaining occupational physical behaviors is associated with a non-significant 42% decreased risk of days with knee pain in the multivariate analysis [RR 0.58 (95% CI (0.32–1.05), P=0.07].

Outcome		Univariate analysis				Multivariate analysis		
		RR	95% CI	P-value		RR	95% CI	P-value
Knee pain days								
	Sedentary_ilr1	1.07	0.75–1.52	0.70		1.06	0.78–1.43	0.71
	Standing_ilr1	1.19	0.58–2.46	0.63		1.47	0.75–2.90	0.26
	LPA_ilr1	1.11	0.79–1.55	0.55		1.10	0.78–1.57	0.58
	MVPA_ilr1	0.71	0.36–1.38	0.31		0.58	0.32–1.05	0.07
Knee pain intensity								
	Sedentary_ilr1	1.01	0.72–1.40	0.97		0.99	0.75–1.31	0.93
	Standing_ilr1	1.14	0.58–2.27	0.70		1.28	0.68–2.40	0.44
	LPA_ilr1	1.12	0.82–1.54	0.47		1.13	0.82–1.56	0.47
	MVPA_ilr1	0.77	0.41–1.45	0.42		0.70	0.41–1.21	0.20

### Days with knee pain

As shown in table 3, none of the reported estimates concerning days with knee pain were statistically significant. However, increasing the occupational time spent in MVPA relative to the other occupational behaviors was associated with a trend towards decreased RR of days with knee pain (0.58, 95% CI 0.32–1.05, P=0.07].

Specifically, figure 2A shows that increasing occupational MVPA by 20 minutes relative to the rest of the occupational behaviors was associated with a non-significant 16% decreased risk of days with knee pain (RR 0.84, 95% CI 0.70–1.02). The CI indicated a large uncertainty, especially for occupational LPA.

### Knee pain intensity

Overall, table 3 and figure 2B show the same trends in the direction of knee pain intensity outcomes as for the days with knee pain outcome, but with smaller magnitudes. Again, all estimates were non-significant.

**Figure 2A:**
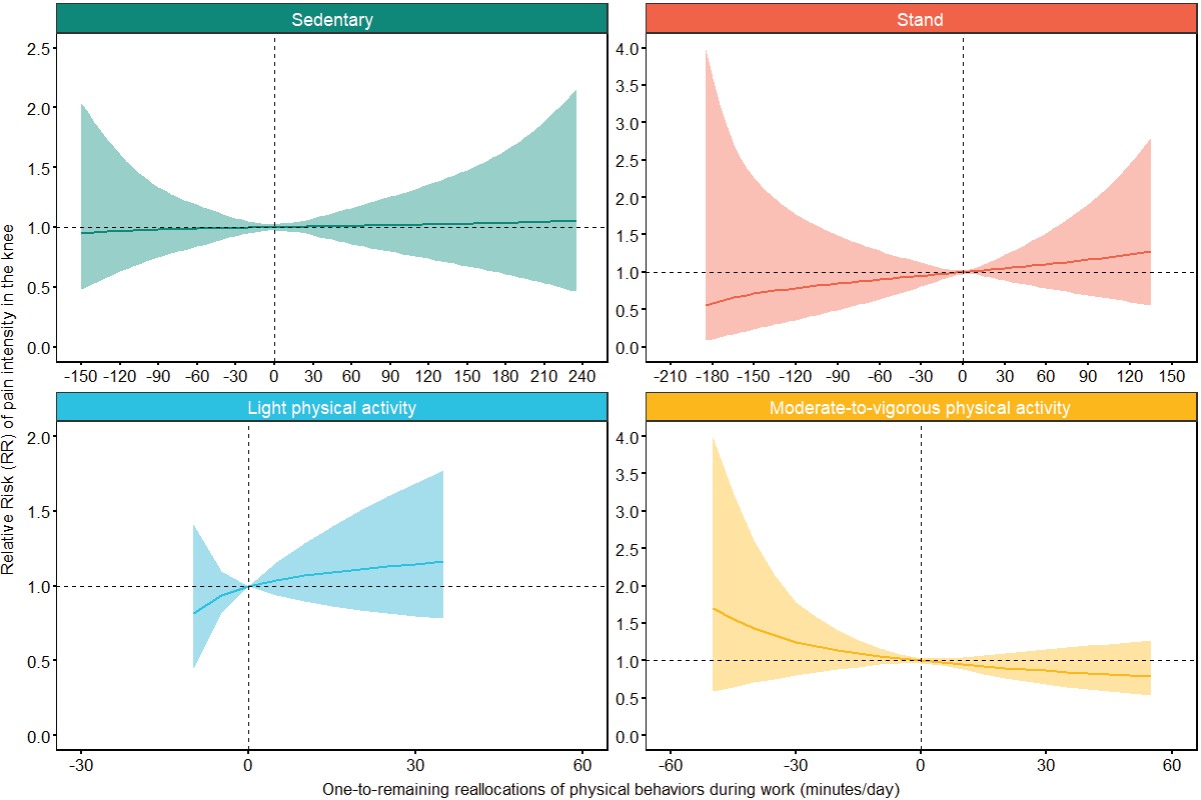
One-to-remaining reallocations of occupational physical behaviors and days with knee pain. Results of a compositional isotemporal substitution analysis based on the negative binomial generalized linear mixed-effects model. “0” on the x-axis represents the average composition of work. Positive values on the x-axis represent the reallocation of minutes to the particular occupational physical behavior from the remaining physical behaviors while negative values represent the reallocation of minutes from the particular occupational physical behavior to the remaining physical behaviors. The y-axis represents the relative risk (RR) of days with knee pain relative to the risk associated with the reference compositions. The ribbons along the lines indicate the 95% confidence intervals of the resulting estimates. [Stand=standing].

**Figure 2B:**
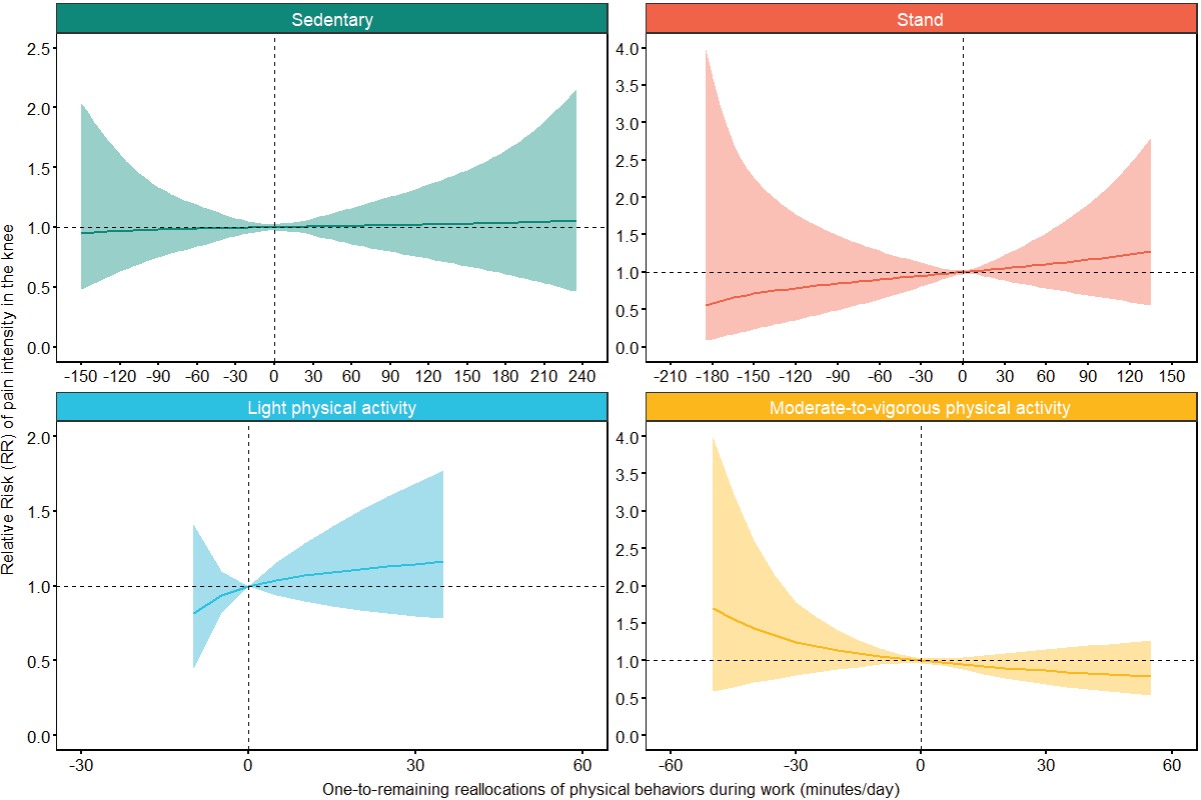
One-to-remaining reallocations of occupational physical behaviors and knee pain intensity. Results of a compositional isotemporal substitution analysis based on the negative binomial generalized linear mixed-effects model. “0” on the x-axis represents the average composition of work. Positive values on the x-axis represent the reallocation of minutes to the particular occupational physical behavior from the remaining physical behaviors while negative values represent the reallocation of minutes from the particular occupational physical behavior to the remaining physical behaviors. The y-axis represents the relative risk (RR) of knee pain intensity relative to the risk associated with the reference compositions. The ribbons along the lines indicate the 95% confidence intervals of the resulting estimates. [Stand=standing].

### One-to-one reallocations

One-to-one reallocation analyses showed that the most marked – though still non-significant – alterations in the risk estimates for knee pain were found by compensatory increasing/decreasing time with occupational LPA or occupational standing by decreasing/increasing time with occupational MVPA (figure 3A–B).

**Figure 3A:**
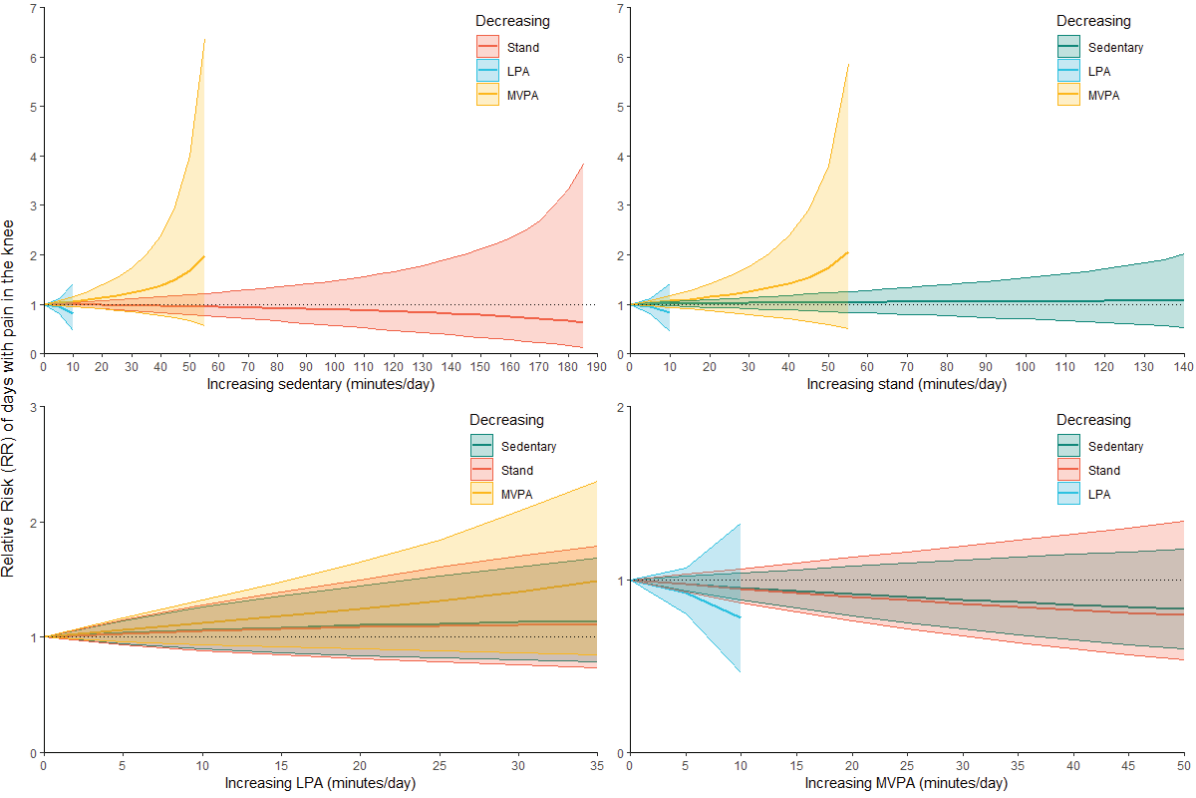
One-to-one reallocations of occupational physical behaviors and days with knee pain. Results of a compositional isotemporal substitution analysis based on the negative binomial generalized linear mixed-effects model. Positive values on the x axis represent the reallocation of minutes to (i.e. increasing time in) the particular occupational physical behavior from (i.e. decreasing time in) another particular physical behavior, while keeping the remaining behaviors constant. “0” on the x axis represents the average composition of work. The y axis represents the relative risk (RR) of days with knee pain relative to the risk associated with the reference compositions. The ribbons along the lines indicate the 95% confidence intervals of the resulting estimates. Stand = standing, LPA = light physical activity, MVPA = moderate-to-vigorous physical activity.

**Figure 3B:**
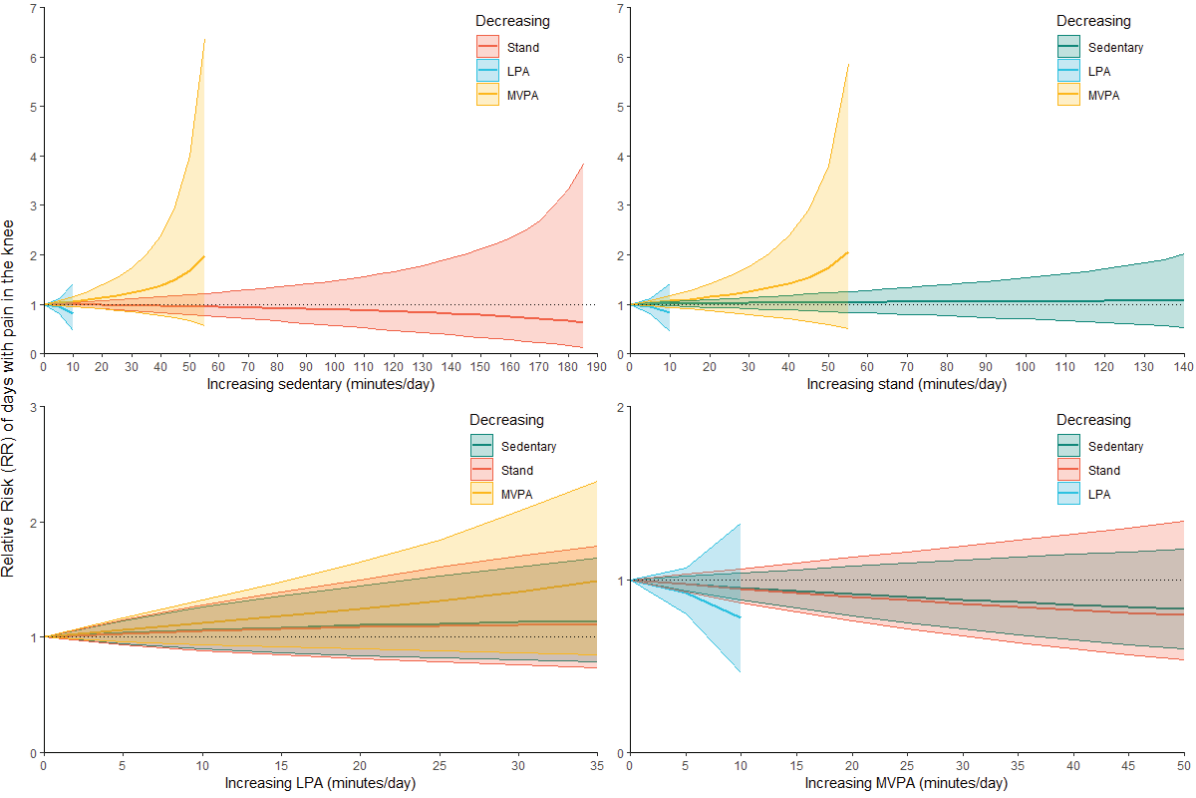
One-to-one reallocations of occupational physical behaviors and knee pain intensity. Results of a compositional isotemporal substitution analysis based on the negative binomial generalized linear mixed-effects model. Positive values on the x axis represent the reallocation of minutes to (i.e. increasing time in) the particular occupational physical behavior from (i.e. decreasing time in) another particular physical behavior, while keeping the remaining behaviors constant. “0” on the x axis represents the average composition of work. The y axis represents the relative risk (RR) of knee pain intensity relative to the risk associated with the reference compositions. The ribbons along the lines indicate the 95% confidence intervals of the resulting estimates. Stand = standing, LPA = light physical activity, MVPA = moderate-to-vigorous physical activity.

### Sensitivity analysis

In total, 145 of 377 (38%) reported a knee pain intensity of ≥3 and were therefore considered knee pain cases, whereas the remaining 232 (62%) were classified as non-knee pain cases.

Overall, the same trends were seen in the sensitivity analyses as in the main analyses. All results were statistically non-significant. Multivariate models among both knee-pain and non-knee-pain cases showed that increasing occupational time spent in MVPA consistently though statistically non-significantly decreased the risk of days with knee pain and knee pain intensity, with lower RR estimates compared to the risk estimates from the main analyses including all participants. Specifically, a trend was found for increasing MVPA and reduced knee pain intensity (RR 0.36, 95% CI 0.12–1.13, P=0.08) among non-knee pain cases.

## Discussion

This prospective study investigated the association between baseline accelerometer-measured occupational physical behaviors and the risk of knee pain measured one year later among eldercare workers. Although no statistically significant associations were found, some trends were observed in the data. Specifically, increasing occupational time spent in MVPA showed a non-significant trend (P=0.07) towards reducing the risk of days with knee pain in the main analysis, while also showing a non-significant trend (P=0.08) towards reduced knee pain intensity among non-knee pain cases in the sensitivity analysis.

### Comparison to other studies

To our knowledge, the cross-sectional study by Locks et al (17) remains the only previous study investigating the association between technically measured, ie, by accelerometry, occupational physical workload, and the risk of knee pain. Additionally, their study investigated occupational standing, one of the occupational physical behaviors also included in the present study. Locks and colleagues (17) reported that frequent short bouts (0–5 minutes) – but not total duration – of accelerometry-measured occupational standing were associated with an increased knee pain intensity, also among those with higher knee pain intensity (≥3/10 on a 0–10 NRS) (17). The present study contributes to the existing literature by demonstrating that an increase in accelerometry-measured total duration of occupational standing, relative to other occupational behaviors, was associated with a non-significant elevation in the risk of days with knee pain (RR 1.47, 95% CI 0.75–2.90, P=0.26) and knee pain intensity (RR 1.28, 95% CI 0.68–2.40, P=0.44) among eldercare workers. Notably, the effect sizes for these associations were larger than those observed for increased occupational sedentary time or occupational LPA. However, given the lack of statistical significance, these findings should be interpreted with caution, as they may simply be attributable to chance. Future research should aim to confirm or reject these preliminary and uncertain findings and thereby advance our understanding of occupational standing in terms of the risk of knee pain.

Previous research, primarily based on self-reported measures of occupational physical activity, has shown mixed results (10–16). Some studies have found an increased risk of non-specific knee pain or knee osteoarthritis related to increased self-reported occupational standing (16, 38), walking (16, 38), or climbing (14, 16), whereas other similar studies have not found any associations (13, 15). Thus, our findings suggesting a *decreased* risk of knee pain with increasing occupational MVPA could seem in contrast to the literature showing an *increased* risk of knee osteoarthritis with higher exposure to occupational walking and stair climbing (14, 16). Notably, however, our accelerometry-measured MVPA category primarily captures dynamic whole-body behaviors like walking and stair climbing, but our single-sensor thigh-based accelerometer setup does not allow for capturing possibly co-occurring knee-straining behaviors or postures such as lifting or carrying, or kneeling or squatting, and the MVPA category may therefore reflect lower knee joint loading. In fact, our finding regarding occupational MVPA seems more in alignment with research on leisure-time physical activity and exercise, where both observational (39, 40) and interventional (41, 42) evidence has suggested a benign/trivial or even positive role of MVPA like walking for knee joint health. One possible explanation for this may be that occupational – similarly to leisure-time – MVPA induces positive physiological adaptations, eg, increased knee extensor strength, a physiological feature previously shown to affect the development and progression of knee disorders like knee osteoarthritis (43). In this context, it should be noted that changes in physical behaviors during (work and) leisure time over the year were not captured in the present study. Such possible changes may have influenced knee pain outcomes either independently or interactively with occupational behaviors, thereby potentially attenuating or amplifying the observed associations. Importantly, self-reported measures of physical behaviors have previously been suggested to be imprecise and biased (19, 21), whereby studies applying technical measures of occupational physical behaviors are warranted, though they also come with some of the above-mentioned limitations. Relatedly, Voinier and colleagues (40) recently performed a CoDa analysis among 4796 adults diagnosed with or at risk of knee osteoarthritis and found that neither more whole-day sedentary time nor time with LPA or MVPA assessed by accelerometers were associated with greater loss of knee joint space width over two years, an indicator of knee osteoarthritis progression.

Knee pain caused by conditions like knee osteoarthritis has a multifactorial biopsychosocial etiology and is influenced by biologic factors of mechanical, inflammatory, and metabolic nature as well as psychosocial factors during work and leisure time (1, 10, 12). Hence, in a work context, effective biopsychosocial prevention of knee pain entails ensuring psychosocial well-being and finding that “sweet-spot” and optimal balance between occupational physical activity-induced physiological stimulus and adequate rest, while also understanding how this interacts with other influences during, eg, leisure time (44, 45).

### Implications and future perspectives

Although the findings of this study were not statistically significant, they suggest potential associations between occupational physical behaviors and knee pain risk that warrant further investigation. Notably, the observed trend for MVPA (P=0.07) provides preliminary evidence that should be confirmed in studies with larger sample sizes and longer follow-up periods, which may detect associations that the present study was unable to due to eg, inadequate statistical power.

To contextualize the potential practical/clinical relevance of our results, the isotemporal substitution analysis indicated that increasing occupational MVPA by 20 minutes was associated with a RR of 0.84 (95% CI 0.70–1.02) for days with knee pain. Applied to the sample’s baseline mean of 4.9 days per month with knee pain, this point estimate equates to a reduction of approximately 0.8 days, from 4.9 to 4.1 days per month. However, the wide CI spans from a potentially meaningful 30% reduction in risk to a slight 2% increase, underscoring the statistical uncertainty of these estimates. Thus, future research with larger samples and repeated long-term follow-ups is needed to clarify these potential associations and their clinical relevance.

If the present findings are confirmed in large-scale studies, intervention studies testing the effects of altered occupational physical behaviors on knee pain outcomes could be instrumental in establishing causal relationships and informing future evidence-based workplace policies aimed at preventing knee disorders and their consequences (18).

### Strengths and limitations

This study had some limitations. First, the study did not utilize repeated measures of the exposure, which could have increased statistical power by capturing periodic variation in occupational physical behaviors over a year and their associations with knee pain. Thus, our results may be confounded by changes in participants’ occupational physical behaviors during the one-year follow-up period. A number of factors – such as seasonal variation in work demands, changes in health status, job tasks, lifestyle, or behavioral adaptations in response to knee pain – could have occurred over time. These changes may introduce considerable variability, potentially influencing the association between the baseline exposure measurement and the outcome. Second, the study may be subject to the healthy worker effect, whereby healthier – ie, selected – individuals are more likely to remain in the workforce, potentially leading to an underestimation of the true associations (46). Importantly, though, when comparing participants from the complete DOSES cohort (26) and those providing accelerometer measurements in the present study, they show similar sociodemographic characteristics, suggesting that the analytic sample in this study is representative of the original cohort to a large degree. Third, a larger sample could have increased the precision and decreased the uncertainty in the estimates. Although we included multiple potential confounders in our models, there is a possible risk of residual confounding from other potentially co-occurring knee-straining postures and behaviors, such as kneeling, squatting, and lifting at work, which have previously been linked to increased risk of knee disorders (10, 12, 15, 16) and which we did not assess in the current study. Another limitation of accelerometry is the inability to capture contextual factors and the intensity and joint loading associated with the performed work.

The study also had important strengths. First, our predictor variable relied on 24-hour accelerometer measurements that have previously shown high sensitivity and specificity (29) and were conducted over several consecutive days, thereby capturing variations in exposure between workdays more effectively than single-day measurements (20). Second, this study addresses a highly understudied subject within occupational health research and applied an advanced analytical technique, ie, compositional data analysis, which addresses some methodological shortcomings associated with other more often-used methods that do not take the compositional nature of physical behaviors into account (23). Third, the longitudinal design with a one-year follow-up on knee pain and multivariate adjustment reduces the risk of reverse causation. Nonetheless, reverse causality cannot be ruled out – not only because pre-existing knee pain may have influenced occupational physical behaviors at baseline, but also because knee pain arising after the accelerometer measurements of occupational physical behaviors could have altered physical behaviors during follow-up, which we did not capture.

Although our findings are not necessarily generalizable to other occupational groups or the general working population, focusing solely on eldercare workers reduced confounding effects from variations in work characteristics and socioeconomic factors between occupational groups.

### Concluding remarks

Our study found no statistically significant associations between occupational physical behaviors measured by accelerometry at baseline and knee pain reported one year later among eldercare workers. However, we observed a non-significant trend suggesting that higher levels of MVPA during work may be associated with reduced knee pain risk, though this requires confirmation in larger studies.
